# The admission neutrophil-to-lymphocyte ratio improves early prediction of poor functional outcome beyond the modified Rankin Scale in viral encephalitis: a cohort study

**DOI:** 10.3389/fneur.2026.1948837

**Published:** 2026-10-15

**Authors:** Jingxuan Fu, Dequan Tian, Bowen Wang, Yanyan Wu, Wenna Liu, Diandian Chen, He Lan, Can Wang, Peichang Wang, Jingrong Cao

**Affiliations:** 1 Department of Clinical Laboratory, Xuanwu Hospital, Capital Medical University, Beijing, China; 2 Department of Medical Laboratory Technology, Capital Medical University, Beijing, China

**Keywords:** incremental value, modified Rankin Scale, neutrophil-to-lymphocyte ratio, nomogram, prognosis, viral encephalitis

## Abstract

**Background:**

The admission modified Rankin Scale (mRS) strongly predicts outcome in viral encephalitis (VE) but does not capture the systemic inflammatory response of severe disease. We tested whether the neutrophil-to-lymphocyte ratio (NLR) improves early prediction of poor functional outcome beyond the admission mRS.

**Methods:**

We studied 200 patients admitted with VE at a single center; the primary outcome was a poor functional outcome at discharge (mRS 3-6). Among routine admission blood tests we identified the markers most strongly associated with poor outcome and selected the leading one, the NLR, by LASSO logistic regression. Incremental value beyond the admission mRS was assessed primarily by a likelihood-ratio test (LRT) for the nested models and supported by the change in discrimination (ΔAUC), reclassification, and decision curve analysis. We predefined a stratified analysis of whether the NLR predicted outcome within bands of admission mRS. The final model was internally validated with 1000 bootstrap resamples, without repeating the initial marker selection.

**Results:**

Forty patients (20.0%) had a poor outcome. After correction, 12 of 16 candidate variables were associated with poor outcome, dominated by inflammatory indices. The admission mRS alone discriminated poor outcome with an apparent AUC of 0.884; adding NLR raised it to 0.912. The gain was significant by LRT (*χ*^2^ = 8.5, *p* = 0.004; NLR odds ratio 1.14 per unit, 95% CI 1.03–1.25), with a ΔAUC of 0.029 (95% CI 0.012–0.046). In the mRS 3–5 stratum, where 39/40 poor outcomes occurred, NLR remained incrementally predictive (*n* = 133; odds ratio 1.15, 95% CI 1.04–1.27, *p* = 0.007). The two-variable model was well calibrated (bootstrap-corrected slope 0.97) and showed greater net benefit than the mRS alone.

**Conclusion:**

In VE, adding the admission NLR to the admission mRS modestly but significantly improves early prediction of poor outcome at discharge, with the increment persisting within admission-severity strata (mRS 3–5). A simple two-variable nomogram (a development-stage tool) is presented for early in-hospital risk flagging. Further analysis suggested that this gain was primarily driven by increased confidence in identifying patients with favorable outcomes, rather than by earlier detection of those who would eventually have poor prognosis.

## Introduction

Viral encephalitis (VE) is a major cause of infectious neurological morbidity and mortality (1), and herpes simplex virus remains the leading cause of sporadic, potentially fatal encephalitis worldwide (2). Even with timely antiviral therapy, a substantial fraction of survivors are left with lasting functional disabilities and cognitive sequelae. Identifying which patients are at risk of a poor outcome early in the admission process is therefore a clinical priority, as it informs monitoring intensity and the threshold for escalating care.

The modified Rankin Scale (mRS) recorded at admission summarizes initial neurological severity and is one of the strongest and most accessible predictors of functional outcome. Most established prognostic factors in acute encephalitis are largely reflected in the admission neurological assessment scale (3). What the mRS does not capture is the systemic inflammatory response that accompanies severe encephalitis. Clinicians consequently lack an inexpensive, universally available marker that enhances prognosis beyond the initial clinical examination.

The neutrophil-to-lymphocyte ratio (NLR), obtained from the routine admission complete blood count, is a simple index of the balance between innate inflammatory activation and adaptive immune competence. An elevated NLR predicts poor functional outcomes and mortality in acute ischemic and hemorrhagic stroke (4) and tracks disease severity and treatment response in autoimmune encephalitis (5). In VE specifically, the evidence is thinner: peripheral NLR has been reported to associate with clinical outcomes (6), and machine-learning analyses of severe Japanese encephalitis have ranked it among the most informative prognostic features (7). These studies establish that NLR is associated with outcomes. However, whether the NLR provides additional prognostic value beyond the admission mRS remains largely unknown.

We therefore set out to build a prognostic tool that can be applied within hours of admission and that enhances outcome prediction beyond the admission mRS alone. Using a cohort of patients with viral encephalitis defined by consensus criteria, we screened early blood markers for their association with poor outcomes, investigated whether the most informative of them added discrimination and clinical net benefit once the admission mRS was accounted for, confirmed that any gain survived within admission-mRS strata, and rendered the resulting model as a development-stage bedside nomogram.

## Materials and methods

### Study design and participants

This was a retrospective single-center cohort study of consecutive patients (≥14 years) admitted with viral encephalitis at Xuanwu Hospital, Capital Medical University, between January 2021 and December 2025. The final analytic cohort comprised 200 patients. The study was approved by the institutional ethics committee. All patients met the case definition of the International Encephalitis Consortium (8). Alternative infectious, autoimmune, metabolic, and other encephalopathies were excluded. Inclusion criteria: (1) age ≥14 years; (2) fulfillment of the diagnostic criteria for definite or probable viral encephalitis; (3) hospitalization at our center with a completed CSF examination. Exclusion criterion: absence of a documented modified Rankin Scale (mRS) score at admission or discharge. The patient flow is shown in Supplementary Figure S1.

### Predictors

We extracted demographic data, the admission mRS, and routine blood tests obtained during the early phase of admission. Sixteen candidate predictors were predefined for screening: two demographic variables (age and sex) and 14 routine admission blood markers. We predetermined that the first variable to enter the LASSO regularization path would be the key predictor for further investigation. The 14 blood markers comprised the complete blood count and its derived indices (neutrophil count, lymphocyte count, monocyte count, platelet count, neutrophil percentage, and the NLR); serum biochemistry (albumin, lactate dehydrogenase [LDH], glucose, potassium, sodium); C-reactive protein (CRP); and two composite indices, the neutrophil-percentage-to-albumin ratio (NPAR) and the pan-immune-inflammation value (PIV). The admission mRS was not part of this screening pool; it served as the reference predictor against which the incremental value of the selected marker was assessed. The three composite inflammatory indices were calculated as follows: the NLR as the absolute neutrophil count divided by the absolute lymphocyte count; the NPAR as the neutrophil percentage divided by the serum albumin concentration (g/dL); and the PIV as the product of the neutrophil, monocyte, and platelet counts divided by the lymphocyte count (all absolute counts, 10^9^/L). The admission mRS was assigned by the treating neurologist at presentation. The complete blood count and biochemistry used here were the first set obtained after admission, drawn during the initial diagnostic work-up. Five variables had missing values: CRP (*n* = 9, 4.5%), LDH (*n* = 4, 2.0%), and serum glucose, potassium, and sodium (*n* = 1 each, 0.5%); the remaining 11 candidate variables were complete. For LASSO selection, which requires a complete design matrix, the few missing values were replaced with the variable median. Both predictors retained in the final model (admission mRS and NLR) were complete for all 200 patients.

### Outcome

The primary outcome was a poor functional outcome at discharge, defined as mRS 3–6 versus 0–2. This dichotomy separates functional dependence or death (mRS 3–6) from functional independence (mRS 0–2) and is the standard outcome definition in stroke and encephalitis cohorts (9). We report in-hospital death (mRS 6) separately. We frame the resulting tool as an early in-hospital risk flag rather than a predictor of long-term disability. Discharge mRS was assessed by trained neurologists. The assessment was performed prospectively, and the assessors were blinded to laboratory results.

### Statistical analysis

Continuous variables were reported as median (interquartile range [IQR]) and compared using the Mann–Whitney *U* test; categorical variables were compared with the chi-square test. Each candidate variable was screened against the primary outcome, and *p*-values were adjusted for multiple comparisons using the Benjamini–Hochberg false-discovery-rate (FDR) procedure; *q* < 0.05 was considered significant. Both final-model predictors (admission mRS and NLR) were complete in all 200 patients.

To select a parsimonious set of markers while limiting overfitting, LASSO logistic regression was applied to all 16 standardized candidate predictors. The L1 penalty (*λ*) was tuned using 5-fold stratified cross-validation, with the cross-validated area under the ROC curve as the selection criterion. The cross-validation–optimal penalty was *λ* = 1.37, at which the mean cross-validated AUC was 0.806, and 11 of the 16 candidate markers were retained. Because the cross-validation–optimal λ retained a relatively large set, and because the effective number of events per variable at the selection stage was low (40 events across 16 candidates ≈ 2.5), we inspected the LASSO selection path—the order in which markers entered as the penalty was relaxed—and included only the leading marker(s) in the nested model comparison. We predefined parsimony as the primary model-building principle: the simplest model that retained a significant incremental term over the admission mRS was preferred, rather than selecting based on calibration-test *p*-values.

Incremental prognostic value over the admission mRS was assessed using a predefined hierarchy of evidence. The primary test was the likelihood-ratio test (LRT) for the nested logistic models (admission mRS vs. admission mRS + marker), which is the appropriate test for nested models and avoids the known conservatism of the DeLong test in this setting (10). Supporting evidence included: (i) the change in AUC (ΔAUC), with 95% confidence intervals obtained from 2000 bootstrap resamples, and the DeLong test reported secondarily for continuity with prior literature (11); (ii) reclassification, reported as the event and non-event components of the continuous net reclassification index (NRI) with bootstrap confidence intervals, along with a single summary NRI for comparability, as the summary statistic is unbounded and prone to overstatement (12); and (iii) the integrated discrimination improvement (IDI) (13). Discrimination and calibration were internally validated using 1,000 bootstrap resamples (Harrell optimism correction) (14); apparent values, mean optimism, and optimism-corrected values were reported together on a single scale. The resampling was applied to the fixed final model. Calibration was summarized by the calibration slope and intercept (bootstrap-corrected) and by the Hosmer–Lemeshow test, the latter reported descriptively only due to its known low power at this sample size. The linearity of the admission mRS term was tested by comparing the linear specification against a categorical (dummy-coded) specification via LRT. The linearity of the NLR was tested in the same manner, by comparing the linear specification against a restricted cubic spline with three knots placed at the 10th, 50th, and 90th percentiles using LRT. Clinical utility was assessed through decision curve analysis (15), and an operating threshold with sensitivity, specificity, and predictive values was reported. Because the admission mRS and the discharge outcome are recorded on the same instrument, we predefined a stratified analysis to test the NLR effect within admission-mRS bands. A nomogram was derived from the final model. Analyses were performed in Python 3.14.3; a two-sided *p* < 0.05 was considered significant.

## Results

### Cohort characteristics

As shown in Table 1, a total of 200 patients with VE were included (median age 43.0 years; 65.0% male). Disease was severe at presentation, with a median admission mRS of 3.0 (IQR 2.0–4.0). mNGS identified a causative pathogen in 156 patients (78.0%), most commonly HSV-1 (22.0%), EBV (21.0%), and VZV (11.5%). The median length of stay was 15.0 days. At discharge, 160 patients (80.0%) had a favorable outcome (mRS 0–2) and 40 (20.0%) had a poor outcome (mRS 3–6); the poor-outcome group included 2 in-hospital deaths (mRS 6).

**Table 1:** Baseline characteristics of the cohort, overall and stratified by functional outcome at discharge.

Variable	Overall (*n* = 200)	mRS 0–2 (*n* = 160)	mRS 3–6 (n = 40)	*p*-value
Age, y, median (IQR)	43.0 (30.8–56.2)	43.0 (31.0–56.0)	38.0 (27.0–61.2)	0.910
Male sex, *n* (%)	130 (65.0)	111 (69.4)	19 (47.5)	0.010
Admission mRS, median (IQR)	3.0 (2.0–4.0)	3.0 (2.0–4.0)	5.0 (4.0–5.0)	<0.001
mNGS pathogen, *n* (%)				
HSV-1	44 (22.0)	29 (18.1)	15 (37.5)	0.008
HSV-2	1 (0.5)	1 (0.6)	0 (0.0)	-
VZV	23 (11.5)	21 (13.1)	2 (5.0)	-
EBV	42 (21.0)	32 (20.0)	10 (25.0)	0.487
Mixed	22 (11.0)	20 (12.5)	2 (5.0)	-
Other	24 (12.0)	17 (10.6)	7 (17.5)	-
Negative	44 (22.0)	40 (25.0)	4 (10.0)	-
NLR, median (IQR)	3.5 (2.0–6.1)	2.7 (1.9–4.9)	6.8 (4.3–8.9)	<0.001
Albumin, g/L, median (IQR)	37.4 (34.2–40.1)	37.7 (35.1–40.6)	35.5 (30.6–37.8)	<0.001
Length of stay, d, median (IQR)	15.0 (10.0–20.0)	14.0 (10.0–17.0)	24.0 (12.8–35.0)	<0.001
Rescue during admission, *n* (%)	66 (33.0)	33 (20.6)	33 (82.5)	<0.001
Rescue count, median (IQR)	0.0 (0.0–1.0)	0.0 (0.0–0.0)	4.0 (1.0–7.2)	<0.001
Corticosteroid before admission, *n* (%)	58 (29.0)	42 (26.2)	16 (40.0)	0.118
ICU care before admission, *n* (%)	11 (5.5)	4 (2.5)	7 (17.5)	0.001

Data are presented as median (IQR) for continuous variables and *n* (%) for categorical variables. The poor-outcome group is defined as a discharge mRS of 3–6 (including 2 in-hospital deaths), while the favorable-outcome group is defined as mRS 0–2. *p*-values are derived from the Mann–Whitney *U* test (continuous) or the chi-square test (categorical). “Rescue during admission” is a post-baseline severity descriptor shown for context only; it was not used in any predictive model. IQR, interquartile range; mRS, modified Rankin Scale; mNGS, metagenomic next-generation sequencing; HSV, herpes simplex virus; VZV, varicella-zoster virus; EBV, Epstein–Barr virus; NLR, neutrophil-to-lymphocyte ratio.

Baseline characteristics stratified by outcome are shown in Table 1. The two groups were of similar age (median 38.0 vs. 43.0 years; *p* = 0.91), but fewer men were among patients with a poor outcome (47.5% vs. 69.4%; *p* = 0.010). As expected, the admission mRS was markedly higher in the poor-outcome group (median 5.0 vs. 3.0; *p* < 0.001). The distribution of detected pathogens differed between groups, with HSV-1 overrepresented among patients with a poor outcome (37.5% vs. 18.1%, *p* = 0.008). Patients with a poor outcome also had a substantially higher NLR (median 6.8 vs. 2.7) and lower serum albumin (35.5 vs. 37.7 g/L; both *p* < 0.001), and they had a longer hospital stay (median 24.0 vs. 14.0 days; *p* < 0.001). The “Rescue during admission” descriptor (in-hospital escalation) was also significantly more frequent in the poor-outcome group; as a post-baseline severity marker, it is shown for description only and was not used in any model. Before admission, 58 patients (29.0%) had received corticosteroid therapy and 11 (5.5%) had received ICU-level care (Table 1). Neither exposure was associated with a detectable shift in the admission NLR: median NLR was 3.77 (IQR 2.00–6.25) in corticosteroid-exposed versus 3.54 (IQR 2.05–5.98) in unexposed patients (*p* = 0.45), and 5.20 (IQR 2.11–9.24) versus 3.54 (IQR 2.01–6.05) for prior ICU care versus none (*p* = 0.31).

### Univariate screening with FDR correction

After Benjamini–Hochberg correction, 12 of the 16 candidate variables remained significantly associated with poor outcome (*q* < 0.05; Table 2). The strongest signals were the composite inflammatory indices and neutrophil-related measures: PIV, NLR, neutrophil count, and NPAR were all significant at *q* < 0.001, with PIV more than doubling in the poor-outcome group (median 656.96 vs. 292.19) and NLR rising from 2.74 to 6.84. Serum albumin was the leading inverse correlate (35.5 vs. 37.7 g/L; *q* = 0.002), while LDH and glucose were both elevated (*q* = 0.002). Neutrophil percentage, lymphocyte count, CRP, male sex, and monocyte count were also significant (*q* ≤ 0.035), whereas sodium, platelet count, age, and potassium were not (all *q* > 0.4).

**Table 2:** Univariate association of the 16 candidate variables with poor functional outcome, with false-discovery-rate correction.

Variable	mRS 0–2	mRS 3–6	*p*-value	*q*-value	Sig
Pan-immune-inflammation value (PIV) (*n* = 200)	292.19	656.96	<0.001	<0.001	*
Neutrophil-to-lymphocyte ratio (NLR) (*n* = 200)	2.74	6.84	<0.001	<0.001	*
Neutrophil count, ×10^9^/L (*n* = 200)	4.42	6.84	<0.001	<0.001	*
Neutrophil-percentage-to-albumin ratio (NPAR) (*n* = 200)	18.13	22.23	<0.001	<0.001	*
Serum albumin, g/L (*n* = 200)	37.7	35.5	<0.001	0.002	*
Lactate dehydrogenase, IU/L (*n* = 196)	193.00	250.00	<0.001	0.002	*
Serum glucose, mmol/L (*n* = 199)	5.05	5.82	<0.001	0.002	*
Neutrophil percentage, % (*n* = 200)	68.1	78.9	0.002	0.004	*
Lymphocyte count, ×10^9^/L (*n* = 200)	1.52	1.08	0.003	0.005	*
C-reactive protein, mg/L (*n* = 191)	4.00	11.00	0.004	0.006	*
Male sex, % (*n* = 200)	69.4	47.5	0.010	0.014	*
Monocyte count, ×10^9^/L (*n* = 200)	0.39	0.48	0.026	0.035	*
Sodium, mmol/L (*n* = 199)	140.00	140.60	0.365	0.449	
Platelet count, ×10^9^/L (*n* = 200)	232.50	246.50	0.495	0.566	
Age, y (*n* = 200)	43.00	38.00	0.910	0.964	
Potassium, mmol/L (*n* = 199)	3.91	3.96	0.964	0.964	

Values are the median in each outcome group. *p*-values are unadjusted; *q*-values are Benjamini–Hochberg false-discovery-rate–adjusted *p*-values. An asterisk (*) denotes *q* < 0.05. Markers are ordered by ascending *q*-value. Age and sex are demographic candidates; the remaining 14 are routine admission blood markers. PIV, pan-immune-inflammation value; NLR, neutrophil-to-lymphocyte ratio; NPAR, neutrophil-percentage-to-albumin ratio; CRP, C-reactive protein; LDH, lactate dehydrogenase.

### Marker selection and final model

At the cross-validation-optimal penalty, LASSO retained 11 markers (full standardized coefficients in Table 3). The selection path (Table 4) showed that NLR was the first marker to enter and remained selected as the penalty relaxed, followed by neutrophil count, LDH, and albumin. The apparent discordance between the univariate ranking and the penalized coefficients is explained by collinearity among the neutrophil-derived indices (Supplementary Table S1): PIV, which is arithmetically constructed from the neutrophil, monocyte, and platelet counts divided by the lymphocyte count, was strongly correlated with the terms retained by LASSO (Spearman *ρ* = 0.78 with neutrophil count and *ρ* = 0.75 with NLR). Under an L1 penalty, this shared signal is distributed across the correlated terms, so the near-zero standardized coefficient for PIV reflects redundancy with the retained markers rather than an absence of association with the outcome. Because the number of events per variable was too low to support a multivariable model of this size (40 events; ≈20 events per variable for a two-variable model, but only ≈2.5 at the 16-candidate selection stage), and following our predefined parsimony principle, we carried the single leading marker—NLR—forward and anchored it on the admission mRS. For transparency, we also fit three richer candidates as sensitivity analyses: (i) mRS + albumin + NLR, (ii) mRS + albumin + sex + NLR, and (iii) mRS + NLR + HSV-1 (Table 5). In the final two-variable model, both the admission mRS (odds ratio [OR] 5.87, 95% CI 3.24–10.61; *p* < 0.001) and NLR (OR 1.14 per 1-unit increase, 95% CI 1.03–1.25; *p* = 0.008) were independently associated with poor outcome (Table 5). The linear specification of the admission mRS was adequate: a categorical specification did not fit better (LRT *χ*^2^ = 1.45, df = 4, *p* = 0.84). The linear specification of NLR was likewise adequate: a restricted cubic spline with three knots (at NLR 1.46, 3.55, and 9.90) did not fit better than the linear term (LRT *χ*^2^ = 1.12, df = 1, *p* = 0.29). Because a one-unit increment is small relative to the observed spread of NLR, we also express the same effect on an interquartile-range scale: across the interquartile range of 4.06 units (2.01 to 6.07), the odds of a poor outcome increased 1.69-fold (95% CI 1.15–2.50), approximately the contrast between the favorable-outcome and poor-outcome medians (2.7 vs. 6.8). Refitting the model with log2-transformed NLR gave an odds ratio of 1.80 (95% CI 1.20–2.71) per doubling, with essentially unchanged fit and discrimination. Adjusting for age left the NLR estimate essentially unchanged (OR 1.15 per 1-unit increase, 95% CI 1.04–1.27; *p* = 0.006), although age was weakly correlated with NLR (Spearman *ρ* = 0.17, *p* = 0.014); age itself was not associated with poor outcome (OR 0.99, 95% CI 0.96–1.02; *p* = 0.40) and adding it did not improve the model (LRT *χ*^2^ = 0.71, df = 1, *p* = 0.40). At this event count, LASSO was used to rank and prioritize the candidate markers rather than to fix a final model directly; deliberately limiting the model to the first-entering marker guards against overfitting and yields a parsimonious, interpretable tool whose incremental value over the admission mRS can then be tested rigorously. Because NLR is known to be responsive to corticosteroids, we repeated the primary analysis after excluding all 58 patients with any corticosteroid exposure before admission (*n* = 142, 24 poor outcomes). The incremental value of NLR over the admission mRS was preserved and larger than in the full cohort: the AUC increased from 0.868 to 0.908 (ΔAUC +0.040), the adjusted odds ratio for NLR was 1.23 per one-unit increase (95% CI 1.07–1.43, *p* = 0.005), and the likelihood-ratio test for the NLR term remained significant (*χ*^2^ = 10.33, df = 1, *p* = 0.001), with adequate calibration (Hosmer–Lemeshow *p* = 0.15). Full results are given in Supplementary Table S2.

**Table 3:** Standardized LASSO coefficients at the cross-validation–optimal penalty.

Variable	Std. coefficient	Odds ratio (per SD)	Selected
Serum albumin	−0.563	0.57	Yes
Male sex	−0.472	0.62	Yes
NLR	0.438	1.55	Yes
Neutrophil count	0.363	1.44	Yes
Monocyte count	0.278	1.32	Yes
LDH	0.258	1.29	Yes
Sodium	0.192	1.21	Yes
Age	−0.120	0.89	Yes
PIV	−0.056	0.95	Yes
CRP	−0.018	0.98	Yes
Potassium	0.018	1.02	Yes
Lymphocyte count	0.000	1.00	–
Neutrophil percentage	0.000	1.00	–
Platelet count	0.000	1.00	–
Serum glucose	0.000	1.00	–
NPAR	0.000	1.00	–

Coefficients are on the standardized scale (per one standard deviation), so their magnitudes are directly comparable. The odds ratio is per standard deviation. “Selected” marks the 11 markers retained at the optimal penalty; markers with a coefficient of exactly zero were dropped. NLR, neutrophil-to-lymphocyte ratio; LDH, lactate dehydrogenase; PIV, pan-immune-inflammation value; CRP, C-reactive protein; NPAR, neutrophil-percentage-to-albumin ratio. Coefficients are penalized (shrunken) LASSO estimates, not from an unpenalized refit. Magnitudes are therefore not directly comparable between correlated markers.

**Table 4:** LASSO selection path for the 16 candidate variables.

Entry order	Variable	Enters at penalty *λ*	Direction (poor vs. favorable outcome)
1	NLR	25.00	Higher in poor outcome
2	Neutrophil count	21.01	Higher in poor outcome
3	LDH	21.01	Higher in poor outcome
4	Serum albumin	17.61	Lower in poor outcome
5	Male sex	12.39	Lower in poor outcome
6	Monocyte count	7.32	Higher in poor outcome
7	Sodium	6.14	Higher in poor outcome
8	Age	3.62	Lower in poor outcome
9	Potassium	1.51	Higher in poor outcome
10	CRP	1.51	Higher in poor outcome
11	PIV	1.51	Higher in poor outcome
12	Platelet count	1.06	Higher in poor outcome
13	Neutrophil percentage	1.06	Higher in poor outcome
14	Serum glucose	0.89	Higher in poor outcome
15	NPAR	0.44	Higher in poor outcome
16	Lymphocyte count	0.26	Lower in poor outcome

Markers are listed in the order they entered the model as the L1 penalty was relaxed (a larger penalty λ admits fewer markers). The direction column reports each marker’s marginal association with outcome, which is stable across penalty levels. NLR, neutrophil-to-lymphocyte ratio; LDH, lactate dehydrogenase; CRP, C-reactive protein; PIV, pan-immune-inflammation value; NPAR, neutrophil-percentage-to-albumin ratio.

**Table 5:** Multivariable logistic regression: the final model and the three sensitivity models.

Model	Variable	OR	95% CI	*p*-value
Final model (mRS + NLR)	Admission mRS	5.87	3.24–10.61	<0.001
	NLR	1.14	1.03–1.25	0.008
Sensitivity model 1 (mRS + albumin + NLR)	Admission mRS	5.56	3.07–10.08	<0.001
	Serum albumin	0.92	0.83–1.02	0.102
	NLR	1.12	1.02–1.23	0.022
Sensitivity model 2 (mRS + albumin + sex + NLR)	Admission mRS	5.52	2.99–10.18	<0.001
	Serum albumin	0.90	0.81–1.00	0.042
	Male sex	0.36	0.13–0.98	0.045
	NLR	1.12	1.01–1.24	0.030
Sensitivity model 3 (mRS + NLR + HSV-1)	Admission mRS	5.74	3.15–10.46	<0.001
	NLR	1.14	1.03–1.25	0.009
	HSV-1	1.22	0.45–3.30	0.701

Odds ratios (OR) are per 1-unit increase for continuous variables and versus the reference category for sex. The final model (admission mRS + NLR) was predefined on parsimony grounds. HSV-1 was coded as HSV-1 versus all other or undetected pathogens. The three sensitivity models are reported in the Results. OR, odds ratio; CI, confidence interval; mRS, modified Rankin Scale; NLR, neutrophil-to-lymphocyte ratio.

### Incremental prognostic value over the admission mRS

The NLR was right-skewed (median 3.55, IQR 2.01–6.07; range 0.71–29.88). The admission mRS alone discriminated poor outcomes with an apparent AUC of 0.884 (1000-resample optimism-corrected 0.883). Adding NLR raised the apparent AUC to 0.912 (corrected 0.911); the optimism was negligible in both models (≤0.002), suggesting limited overfitting for these low-dimensional models, at least at this validation step. The significance of variable inclusion was assessed using a predefined likelihood-ratio test (LRT), which revealed that adding NLR significantly improved model fit (*χ*^2^ = 8.5, df = 1, *p* = 0.004). A subsequent Wald test confirmed that for each 1-unit increase in NLR as a continuous variable, the OR was 1.14 (*p* = 0.008), with consistent findings across both tests. The gain in discrimination was modest, but its confidence interval excluded zero: ΔAUC 0.029 (95% CI 0.012–0.046) (Figure 1); the secondary DeLong test was concordant (*z* = 2.58, *p* = 0.010). The incremental model was well calibrated (apparent calibration slope 1.00, intercept 0.00; bootstrap-corrected slope 0.97; Hosmer–Lemeshow *χ*^2^ = 8.83, df = 8, *p* = 0.357; Figure 2). Decision curve analysis showed that adding NLR to the admission mRS increased net benefit over the threshold-probability range of approximately 0.28–0.42, most notably around a threshold of 0.30, where the net benefit of the mRS alone dropped sharply but was preserved after NLR was added (Figure 3). Together, these results indicate that the admission NLR contributes incremental prognostic information to the admission mRS, albeit of modest magnitude.

**Figure 1:**
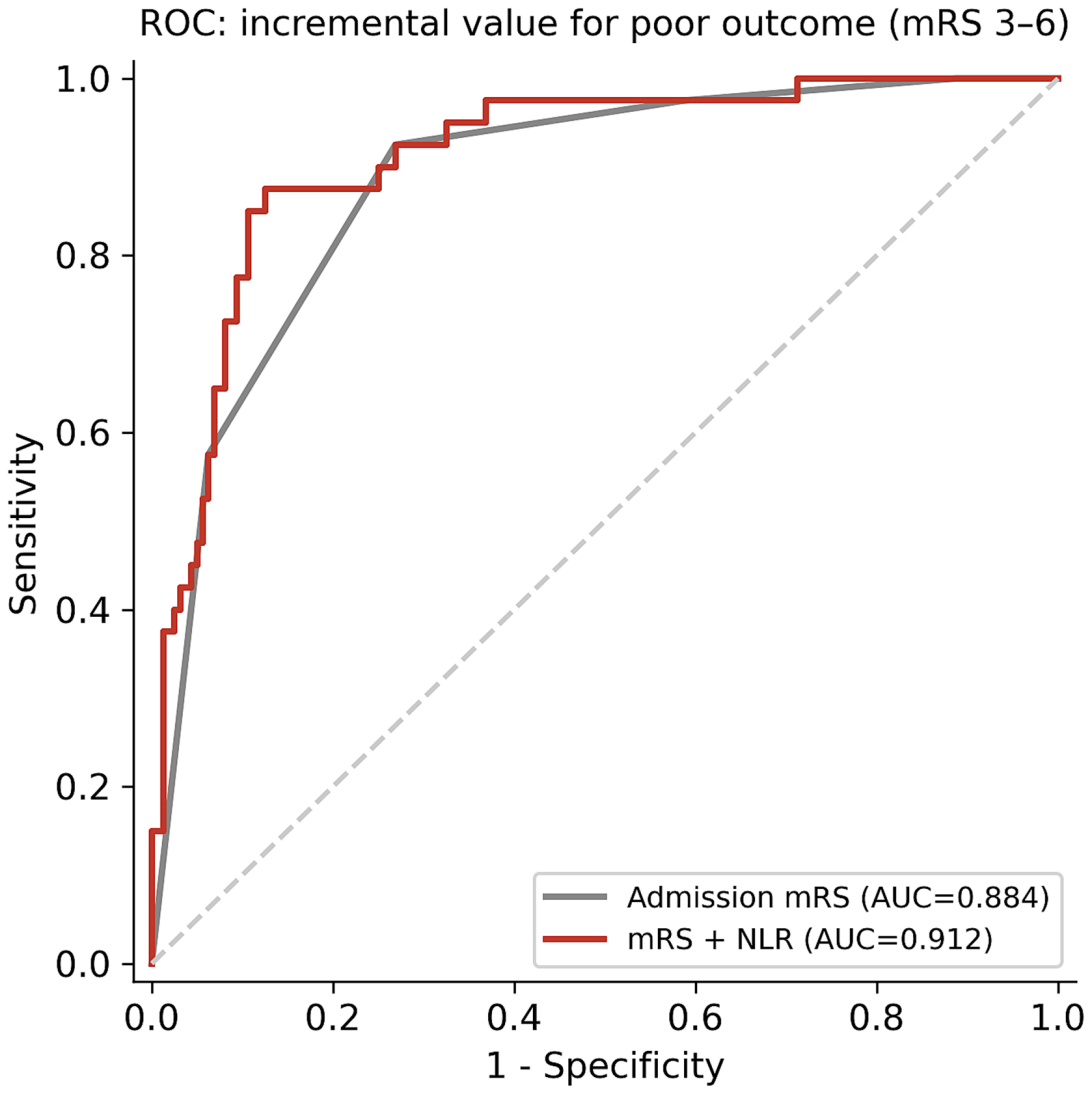
Incremental discrimination of the admission mRS plus NLR over the admission mRS alone. Receiver operating characteristic (ROC) curves for the baseline model (admission mRS alone) and the incremental model (mRS + NLR). The curve for the incremental model lies above that of the baseline model (apparent AUC 0.912 vs. 0.884; ΔAUC 0.029, 95% CI 0.012–0.046; likelihood-ratio test *p* = 0.004), indicating that adding NLR improves discrimination beyond the mRS alone.

**Figure 2:**
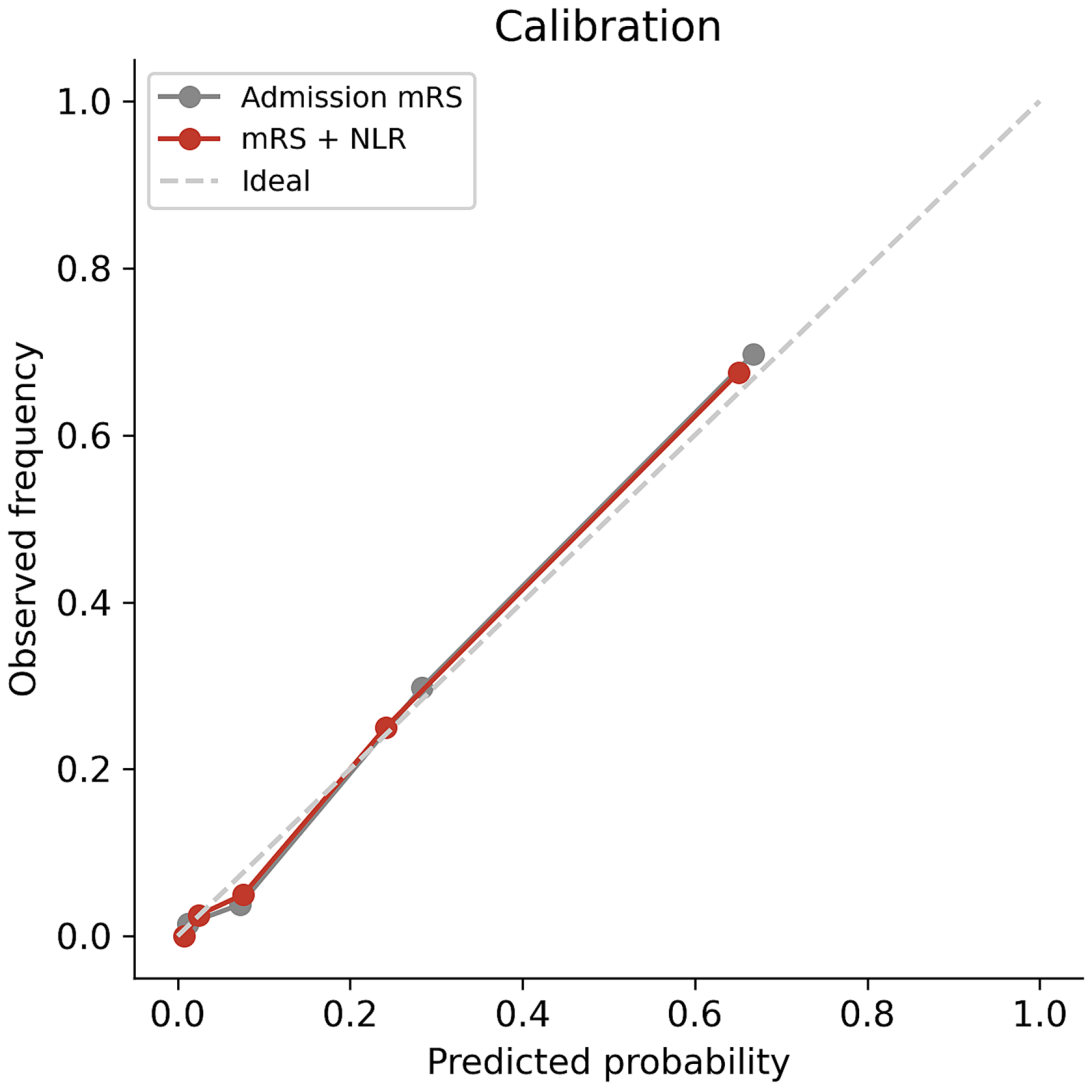
Calibration of the final model. The *x*-axis represents the model-predicted risk of poor outcome, and the *y*-axis shows the observed proportion; points are grouped by predicted-risk band. Perfect calibration lies along the 45° diagonal. The points track the diagonal closely (apparent calibration slope 1.00, intercept 0.00; bootstrap-corrected slope 0.97; Hosmer–Lemeshow *χ*^2^ = 8.83, df = 8, *p* = 0.357).

**Figure 3:**
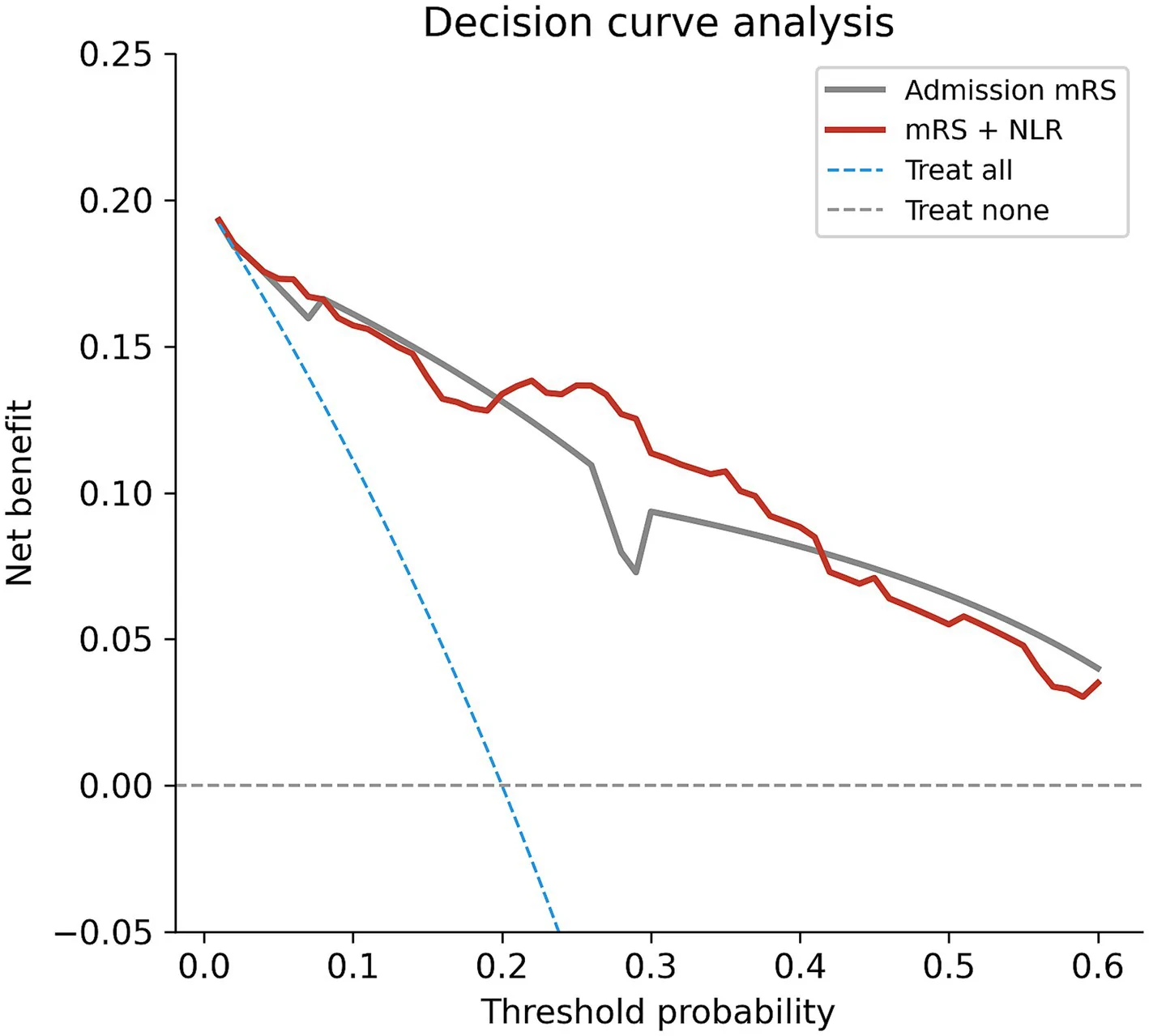
Decision curve analysis comparing the two models with the default strategies. The *x*-axis indicates the threshold probability at which a clinician would act, while the *y*-axis shows the net benefit. The reference strategies are “treat all” and “treat none.” The mRS + NLR curve is positioned above the mRS-alone curve over a threshold-probability range of approximately 0.28–0.42, most notably around a threshold of 0.30.

We assessed whether adding NLR reclassified patients into more accurate risk strata—that is, whether each patient’s predicted risk of a poor outcome shifted in the correct direction once NLR was included. We examined this improvement separately in the two outcome groups rather than as a single combined index because a summary index counts any shift in the correct direction as an improvement, regardless of its magnitude, and is therefore unbounded and prone to overstatement; a group-specific view instead shows whether the benefit is genuine and where it is concentrated. For patients who ultimately had a poor outcome (the event group), we asked whether the model correctly raised the predicted risk; for those who ultimately recovered well (the non-event group), whether it correctly lowered it. The benefit came almost entirely from the non-event group: after NLR was added, patients who ultimately recovered well were correctly assigned a lower predicted risk (non-event NRI + 0.54, 95% CI + 0.41 to +0.67), whereas the improvement in the poor-outcome group was small and non-significant (event NRI + 0.15, 95% CI − 0.14 to +0.46). For comparability with published reports, the continuous NRI combining both components was +0.69 (95% CI + 0.36 to +1.03); as noted above, however, we base our inference on the components rather than on this unbounded summary. Overall discrimination improved only modestly, with a confidence interval that crossed zero (IDI + 0.039, 95% CI − 0.007 to +0.089). Clinically, NLR chiefly strengthened the model’s ability to identify patients with a favorable outcome with confidence (improved specificity) rather than to flag additional patients destined for a poor outcome in advance (sensitivity).

### Incremental value within admission-mRS strata

Because the admission mRS and the discharge outcome are recorded on the same instrument, part of the baseline model’s discrimination reflects autocorrelation rather than genuine prediction: of the 67 patients admitted with mRS 0–2, only 1 (1.5%) had a poor outcome, whereas 39 of the 40 poor outcomes (97.5%) occurred among the 133 patients admitted with mRS 3–5. Therefore, whether NLR can discriminate outcomes within the mRS 3–5 stratum is important, since baseline severity no longer separates the groups in this stratum. Indeed, within this stratum, NLR alone discriminated poor outcomes with an AUC of 0.73, and in a logistic model that also included the admission mRS, the NLR remained independently associated with poor outcomes (OR 1.15 per 1-unit increase, 95% CI 1.04–1.27, *p* = 0.007)—essentially unchanged from the whole-cohort estimate. When both models were rebuilt within this stratum alone, the admission mRS gave an AUC of 0.830, and adding the NLR raised it to 0.879 (ΔAUC +0.049, 95% CI +0.020 to +0.081), consistent with the whole-cohort increment. The NLR therefore carries prognostic information that is not an artifact of the predictor–outcome coupling. Clinically, this is the stratum where prognosis is genuinely in doubt. Patients admitted with mRS 0–2 almost always do well, so the initial examination essentially settles their prognosis; it is the large middle group admitted with mRS 3–5—dependent but alive, and accounting for 39 of the 40 poor outcomes—in whom the bedside picture is ambiguous. That the NLR retains its effect precisely here means it adds information exactly where clinicians most need it.

### Operating characteristics and nomogram

To make the model concrete for clinical discussion, we report its operating characteristics at candidate thresholds (apparent). At a predicted-risk threshold of 0.20, the model flagged 62 of 200 patients and identified poor outcomes with a sensitivity of 0.88, specificity of 0.83, positive predictive value of 0.56, and negative predictive value of 0.96; at a threshold of 0.30, the corresponding values were 0.78, 0.90, 0.66, and 0.94. The high negative predictive value indicates the model is most useful for de-escalation confidence—providing reassurance that a low-risk patient is genuinely low-risk—consistent with the reclassification pattern. A nomogram based on the final model translates the admission mRS and NLR into an individualized probability of poor outcome (Figure 4).

**Figure 4:**
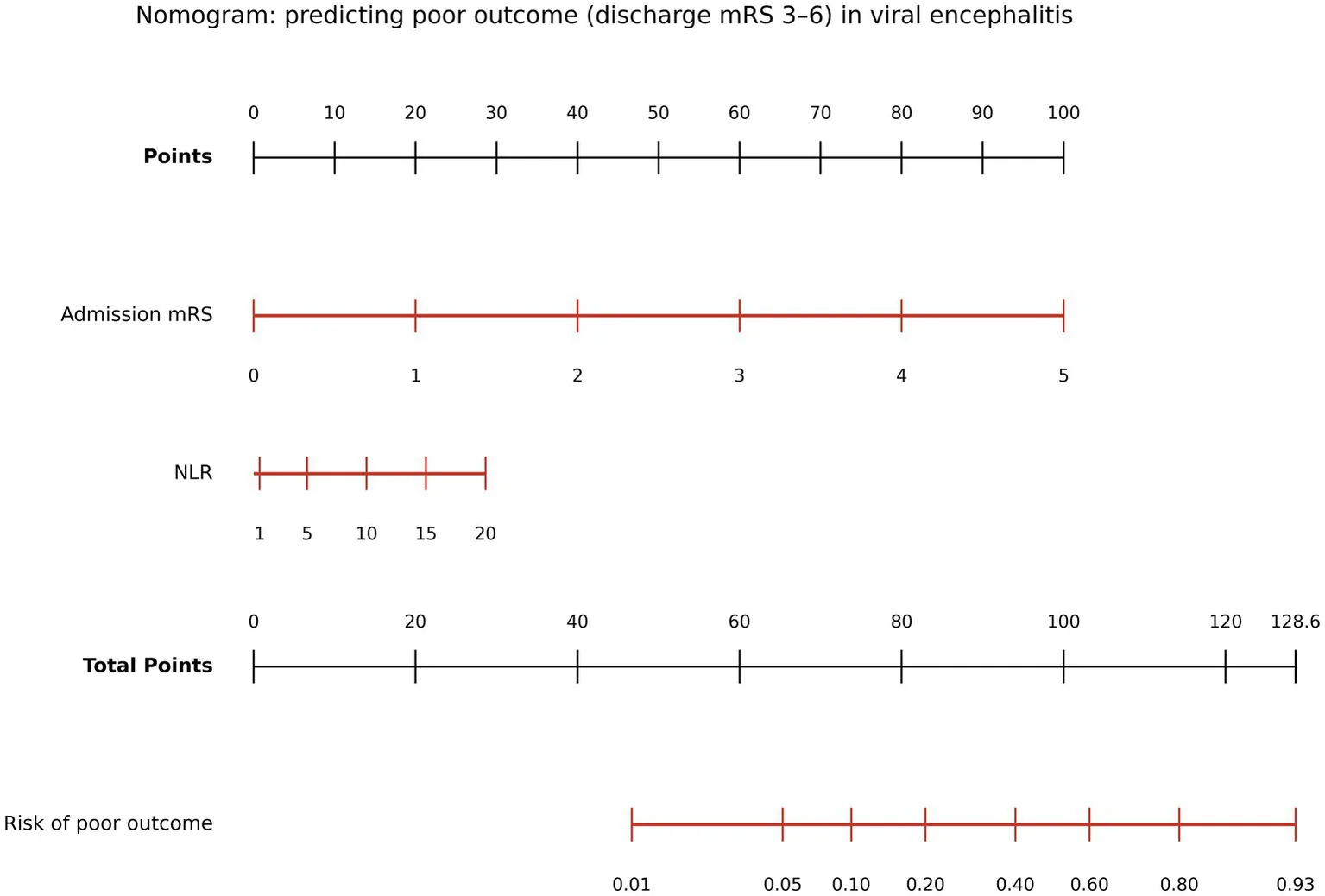
Development-stage nomogram for the final model. Read the points for the admission mRS and the NLR from the top “Points” axis, sum them, and map the total to the predicted probability of poor outcome on the bottom axis.

## Discussion

The main finding of this study is that a routine admission NLR adds prognostic information beyond the admission mRS. Crucially, even among patients with similar admission severity (mRS 3–5), a higher NLR marked those more likely to be functionally dependent at discharge (OR 1.15 per 1-unit increase), so this increment is not merely a re-reading of baseline severity.

Clinically, the value of this increment is to sharpen risk stratification using inputs that are universal and immediate—the initial neurological examination and a routine complete blood count, both available within hours and requiring no specialized infrastructure. This makes it particularly suited to the emergency setting, where an early prognostic signal is most needed yet specialized data are least available. The clinical value of NLR may also apply to other neurological disorders (16, 17). The gain is largest in the moderate-severity “gray zone” (mRS 3–5), where the bedside picture is genuinely uncertain yet almost all poor outcomes arise. In this cohort, the added information was clearest for confidently identifying patients who would do well (negative predictive value 0.94–0.96) and weaker for flagging additional patients who would deteriorate, so the model should support rather than replace clinical judgment. However, no treatment in VE is currently guided by the NLR, so better prediction does not necessarily mean that acting on the score improves outcomes, and because our data came from a large tertiary center, the tool is not yet validated for triage or for primary and low-resource settings.

The decision curve analysis should be interpreted as exploratory. In this analysis, the “treat” decision represents the initiation of an enhanced early surveillance pathway, comprising evaluation for neurological high-dependency or intensive care admission, early or repeated electroencephalography to exclude non-convulsive status epilepticus, shortened intervals between neurological examinations, early swallowing assessment with aspiration and venous thromboembolism prophylaxis, early rehabilitation consultation, and early prognostic discussion with the patient’s family. In this cohort, patients with a low predicted risk rarely had a poor discharge outcome. Whether this observation can safely justify a lower intensity of monitoring cannot be determined from a retrospective single-center dataset and would require prospective evaluation.

Existing outcome-prediction tools in encephalitis are either etiology-specific or derived from ICU-only cohorts, and none is built from variables available at the moment of admission. The NEOS score for anti-NMDAR encephalitis assigns points for ICU admission, treatment delay beyond 4 weeks, lack of clinical improvement within 4 weeks, abnormal MRI, and a CSF white-cell count above 20/μL (18); two of those items can only be determined weeks into the illness. Prognostic modeling in herpes simplex encephalitis has relied on illness-severity scores and the interval to acyclovir (19), and the ICU-derived model of Thakur et al. uses cerebral edema, status epilepticus, and thrombocytopenia (3). Each addresses a narrower population or a later decision point than the present study. Our model requires only the admission mRS and a single ratio from the admission blood count, both available within hours of presentation, and to our knowledge, the incremental value of such a tool over the admission mRS has not previously been reported. Direct comparison of discrimination across these models is not meaningful, because the derivation cohorts, outcome definitions, and prediction horizons differ.

Several limitations temper these conclusions. Most importantly, the NLR is non-specific: critical illness, corticosteroids, and intercurrent infection all raise it. Although some data were available regarding pre-admission corticosteroid use and ICU admission, we lacked detailed information on intensive-care admission, mechanical ventilation, time to antiviral therapy, and pre-sample corticosteroid use (agent, dose, or timing), among other variables, so residual confounding cannot be excluded—“independent of the admission mRS” is not the same as “independent of the clinical drivers of outcome.” Regarding the assessment of mRS, this study has several limitations. The mRS assessments were performed by clinicians during routine clinical practice, and the inherent subjectivity of the mRS may introduce bias. Outcome assessment was based on discharge mRS rather than a fixed time point (90-day mRS was unavailable), resulting in the discharge mRS being captured at different time points along the recovery trajectory of the two groups—patients with favorable outcomes were assessed at an earlier stage, while those with poor outcomes were assessed at a later stage, which may systematically overestimate the differences in prognosis between the groups. Another limitation of this study should be acknowledged. The variable selection process, including univariate FDR screening and LASSO dimensionality reduction, was performed on the complete dataset, whereas the bootstrap optimism correction was applied only to the final regression model. Therefore, this correction does not fully reflect the potential optimism arising from selection uncertainty throughout the entire modeling process. Additionally, because the aim of this study was to establish a rapid and simple model, the effects of different pathogens on the model may not have been adequately examined, and this study only performed a preliminary analysis of HSV-1. Finally, the study was single-center, retrospective, and modest in event count (*n* = 40), which keeps the nomogram in the development stage (TRIPOD type 1b) (20). The absence of external validation is also a limitation, and prospective multicenter validation should be undertaken as future work. These considerations limit the immediate clinical translation of the nomogram. In other words, the NLR cannot serve as a decision-making tool.

The biological rationale for a prognostic role of the NLR in viral encephalitis is sound. The ratio combines two white blood cell types that play opposing roles. When viruses invade the brain, neutrophils are recruited as part of the earliest inflammatory response and increase the permeability of the blood–brain barrier (21). In a mouse model of flaviviral encephalitis, depleting neutrophils improves survival and reduces tissue damage, whereas adoptive transfer of T cells also enhances survival, indicating that the two cell types act in opposing directions (22). Consistently, in patients with tick-borne encephalitis, weaker early virus-specific T-cell responses are associated with more severe disease (23). A high NLR therefore captures a detrimental combination: excessive tissue-damaging inflammation from neutrophils, along with insufficient antiviral T-cell activity. In our cohort, a higher admission NLR was independently associated with poorer functional outcomes. Importantly, the NLR is a systemic blood marker rather than a direct measure of brain inflammation. We interpret it as an index of overall disease severity rather than a specific intracranial pathology.

In conclusion, in viral encephalitis, a routine admission NLR adds modest but incremental prognostic information to the admission mRS, improving early risk stratification with data available at the bedside within hours, and it is most useful in the moderate-severity range where the initial examination is least informative. Further analysis suggested that this gain was primarily driven by increased confidence in identifying patients with favorable outcomes, rather than by earlier detection of those who would eventually have a poor prognosis. Nevertheless, the effect is susceptible to confounding by illness severity and treatment, and the two-variable nomogram remains a development-stage tool. Prospective external validation with a fixed outcome horizon and adjustment for treatment and critical illness covariates is the necessary next step.

## Data Availability

The raw data supporting the conclusions of this article will be made available by the authors, without undue reservation.

## Supplementary material

SUPPLEMENTARY FIGURE S1 Flow of patients through the study.
