# Supplementary material for: The admission neutrophil-to-lymphocyte ratio improves early prediction of poor functional outcome beyond the modified Rankin Scale in viral encephalitis: a cohort study
Source: Front Neurol. 2026 Oct 15;17:1948837. doi: 10.3389/fneur.2026.1948837 (PMC13716551; doi:10.3389/fneur.2026.1948837)
Supplement: Supplementary file 1 [file Table_1.docx]

**Supplementary Tables:**

**Supplementary Table S1.** Spearman rank-correlation matrix for the shortlisted inflammatory indices (n=200).

|  | NLR | Neutrophil count | Neutrophil percentage | Lymphocyte count | Monocyte count | Platelet count | PIV | NPAR |
| --- | --- | --- | --- | --- | --- | --- | --- | --- |
| NLR | 1 | 0.73 | 0.84 | -0.69 | 0.04 | 0 | 0.75 | 0.76 |
| Neutrophil count | 0.73 | 1 | 0.61 | -0.07 | 0.38 | 0.19 | 0.78 | 0.52 |
| Neutrophil percentage | 0.84 | 0.61 | 1 | -0.57 | -0.01 | 0.09 | 0.63 | 0.87 |
| Lymphocyte count | -0.69 | -0.07 | -0.57 | 1 | 0.33 | 0.22 | -0.27 | -0.55 |
| Monocyte count | 0.04 | 0.38 | -0.01 | 0.33 | 1 | 0.19 | 0.56 | 0.04 |
| Platelet count | 0 | 0.19 | 0.09 | 0.22 | 0.19 | 1 | 0.38 | 0.08 |
| PIV | 0.75 | 0.78 | 0.63 | -0.27 | 0.56 | 0.38 | 1 | 0.6 |
| NPAR | 0.76 | 0.52 | 0.87 | -0.55 | 0.04 | 0.08 | 0.6 | 1 |

*Values are Spearman rank-correlation coefficients (ρ) computed in the full cohort of 200 patients. NLR, neutrophil-to-lymphocyte ratio; PIV, pan-immune-inflammation value; NPAR, neutrophil-percentage-to-albumin ratio.*

**Supplementary Table S2.** Sensitivity analysis of the incremental prognostic value of the admission neutrophil-to-lymphocyte ratio, restricted to patients without corticosteroid exposure before admission.

|  | **Full cohort** | **Corticosteroid-unexposed subgroup** |
| --- | --- | --- |
| Patients, n | 200 | 142 |
| Poor outcome (mRS 3–6), n (%) | 40 (20.0) | 24 (16.9) |
| AUC, admission mRS alone | 0.884 | 0.868 |
| AUC, admission mRS + NLR | 0.912 | 0.908 |
| ΔAUC | +0.029 | +0.040 |
| Admission mRS, OR (95% CI) | 5.87 (3.24–10.61) | 4.96 (2.43–10.12) |
| NLR, OR per 1-unit increase (95% CI) | 1.14 (1.03–1.25) | 1.23 (1.07–1.43) |
| *p* for NLR | 0.008 | 0.005 |
| Likelihood-ratio test for NLR, χ² (df = 1) | 8.5 | 10.3 |
| *p* for likelihood-ratio test | 0.004 | 0.001 |
| Hosmer-Lemeshow *p* | 0.36 | 0.15 |

*The Corticosteroid-unexposed subgroup excludes 58 patients with any documented corticosteroid use before admission. Both models are logistic regressions of poor outcome (discharge mRS 3–6) on the admission mRS with and without the admission NLR. Full-cohort values are those reported in the main text. AUC, area under the receiver-operating-characteristic curve; NLR, neutrophil-to-lymphocyte ratio; OR, odds ratio.*
